# An entropy and machine learning based approach for DDoS attacks detection in software defined networks

**DOI:** 10.1038/s41598-024-67984-w

**Published:** 2024-08-06

**Authors:** Amany I. Hassan, Eman Abd El Reheem, Shawkat K. Guirguis

**Affiliations:** Institute of Graduate Studies and Research, Alexandria, Egypt

**Keywords:** Engineering, Electrical and electronic engineering

## Abstract

Software-defined networks (SDNs) have been growing rapidly due to their ability to provide an efficient network management approach compared to traditional methods. However, one of the major challenges facing SDNs is the threat of Distributed Denial of Service (DDoS) attacks, which can severely impact network availability. Detecting and mitigating such attacks is challenging, given the constantly evolving range of attack techniques. In this paper, a novel hybrid approach is proposed that combines statistical methods with machine-learning capabilities to address the detection and mitigation of DDoS attacks in SDN environments. The statistical phase of the approach utilizes an entropy-based detection mechanism, while the machine-learning phase employs a clustering mechanism to analyze the impact of active users on the entropy of the system. The k-means algorithm is used for clustering. The proposed approach was experimentally evaluated using three modern datasets, namely, CIC-IDS2017, CSE-CIC-2018, and CICIDS2019. The results demonstrate the effectiveness of the system in detecting and blocking sudden and rapid attacks, highlighting the potential of the proposed approach to significantly enhance security against DDoS attacks in SDN environments.

## Introduction

SDN is a cutting-edge approach to network management architecture that enhances adaptability, programmability, and responsiveness to dynamic service and application needs. It centralizes the control plane, managing data traffic routing and communication between network parts. Traditional network topologies integrate control planes into routers and switches, while SDN control is centralized in a software-based controller, with control and data planes separated^1^.

The intentional attempt to disrupt traffic on the network is known as a DDoS attack. Disrupting the availability and quality of service (QoS) of essential services by flooding the targeted system’s bandwidth and resources^2^. Attackers exploit SDN's centralized control plane, a single point of failure, to create undetected traffic patterns, making detection and mitigation of DDoS attacks challenging^3^.

Although SDNs are more flexible and manageable, because of their centralized control and programmable nature, they are also vulnerable to DDoS attacks. Due to the dynamic and complicated nature of network traffic patterns and the attackers' constantly changing techniques. It might be difficult to detect and mitigate these attacks in SDN systems.

**Figure 1:**
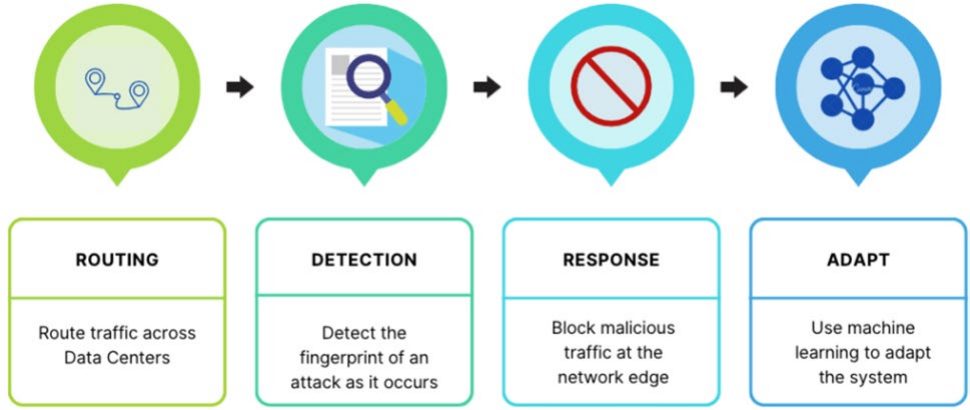
DDoS mitigation stages.

Figure 1 shows the DDoS attack mitigation main steps: traffic routing, attack fingerprint detection, response, and machine learning adaptation^4^. Lately, DDoS attacks have become more challenging to detect due to their variety. For example, multi-vector attacks, where a combination of multiple attack protocols is common. Therefore, more robust defense techniques are required.

The umbrella of DDoS detection and mitigation is wide. It includes several approaches, such as statistical-based ones, that aim to protect networks by analyzing and collecting flow-related statistics^5^. They can be applied in several ways, such as entropy-based techniques, trust management, time-series analysis, and anomaly detection. However, these methods are facing limitations in accurately distinguishing normal and malicious traffic and adjusting to emerging threats. This may lead to potential false positives or false negatives^6^.

Machine learning (ML) algorithms showed a promising potential for detecting and mitigating DDoS attacks^7^. These approaches can analyze network traffic in real time, identify malicious behavior, and adapt detection models based on real-time network data. In addition, they can handle large volumes of network data and detect zero-day attack patterns. It makes them an effective defense against evolving and sophisticated DDoS attacks. On the other hand, ML-based DDoS detection approaches face limitations due to imbalanced datasets and the dynamic nature of attacks^8^. Imbalances can lead to biased behavior and reduced accuracy in recognizing novel attack patterns as discussed by Ullah et al.^9^. Continuous model retraining and adaptation to evolving threats are necessary to maintain efficacy^10^. ML presents an added privilege to traditional detection techniques since there is no single detection method that can provide 100% accuracy^11^.

Blockchain technology is proposed for DDoS detection and mitigation, enhancing network security, scalability, and performance. However, their use in DDoS detection and mitigation faces challenges like potential latency and scalability^12^.

The motivation behind this research is to develop a novel model that improves DDoS attack detection and mitigation in SDN networks by utilizing system entropy and ML clustering techniques. System entropy, a measure of disorder or randomness within a system, presents valuable details regarding the normal behavior of network traffic. Anomalies indicative of DDoS attacks can be identified by tracking variations in entropy levels.

However, depending only on statistical techniques, such as entropy-based detection, could not be accurate or responsive enough, especially in large-scale and dynamic SDN systems^6^. In addition to complementing the statistical method, ML clustering techniques like the K-means algorithm enable the analysis of complex patterns and the identification of anomalous network activity groups.

The combination of system entropy with ML clustering techniques aims to create a comprehensive defense and address several key challenges in DDoS attack detection and mitigation:

- Real-time Detection: The model can identify DDoS attacks in real-time, allowing for quick response and mitigation measures, by continuously monitoring system entropy and utilizing machine learning clustering methods.
- Adaptability: DDoS attack techniques are always changing; therefore, detection systems must adjust and detect new attack patterns. By using machine learning clustering techniques, the model's resilience against new threats is increased since it may dynamically modify its detection skills in response to observed network behavior.
- Scalability: Several network devices and a variety of traffic patterns are common in SDN systems. The suggested approach is made to be scalable to manage the complexity and volume of network traffic, providing accurate detection and extensive coverage throughout the network.

The main contribution of this paper can be highlighted as follows:

1. Surveying the state-of-the-art literature on detecting intrusions in SDN environments and analyzing the research gap.
2. Proposing a novel approach that combines a statistical entropy-based technique with an ML one for the effective detection and mitigation of DDoS attacks. This leads to a comprehensive SDN defense mechanism. This mechanism can mitigate attacks in real time, adapt to evolving attack strategies, and minimize false positives.
3. Conducting an empirical evaluation by utilizing three modern SDN datasets, i.e. CIC-IDS2017, CSE-CIC-2018, and CICIDS2019.
4. Benchmarking with other anomaly detection approaches using three recent public datasets.
5. Reducing false positives and minimizing the impact of false alarms on network operations by combining system entropy with machine learning clustering.
6. Enhancing the efficiency of DDoS detection while improving network resilience and security posture in the face of evolving cyberattacks.

The remaining of this paper is as follows, "Related work" section discusses the related work. "Methods" section demonstrates the proposed methodology for DDoS detection and mitigation. "Evaluation scheme" section provides an in-depth analysis of the evaluation environment used in this study, outlining the dataset, data preprocessing methodologies, and performance metrics for transparent analysis. "Results" section describes the experimental setup and proposed methodology evaluation and compares the results with the state-of-the-art approaches. Finally, the conclusion is provided in Section "Conclusion and future work" in addition to further probable directions for future research.

## Related work

This section examines different defense mechanisms for detecting DDoS attacks. It emphasizes the significance of statistical, machine learning, deep learning, and blockchain approaches.

The survey of the literature on DDoS attacks detection and mitigation techniques, as listed in the introduction section, follows the methodological approaches detailed in the literature review methods outlined in Saied et al^13^.

### Statistical strategies for DDoS detection and mitigation in SDN

Statistical-based attack detection and mitigation methods aim to protect the network by analyzing and collecting flow-related statistics. This is achieved by generating a statistical model for regular traffic to identify malicious flows^5^.

Koay et al.^14^ proposed a novel method to enhance the accuracy of detecting Distributed Denial of Service (DDoS) attacks by introducing a comprehensive set of new entropy-based features. They addressed the limitation of traditional DDoS detection systems, which often rely on a small number of features, leading to certain types of attacks being undetected. To overcome this, they introduced a multi-classifier system that combines multiple entropy-based features with machine learning classifiers. This system improves the generality and accuracy of detecting both low-intensity and high-intensity DDoS attacks. Although this approach may cause slightly longer detection delays due to increased complexity, the trade-off is justified by the system's ability to detect a broader range of DDoS attack types, ultimately enhancing network security.

Tsobdjou et al.^15^ introduced a dynamic entropy threshold technique based on Chebyshev inequality, which offers improved adaptability compared to static criteria across different online scenarios. Through comparative tests, their approach showed superior performance in adjusting to changing conditions, making it promising for dynamic environments.

In a separate study, Sahoo et al.^16^ proposed a method for detecting attacks on the controller by utilizing defined entropy and information distance to identify low-rate DDoS attacks. While this method effectively detected low DDoS attack traffic rates, it encountered challenges in identifying high traffic rates due to variations in network traffic flow. Despite this limitation, the proposed method contributes significantly to DDoS attack detection, especially in scenarios involving low-rate attacks.

P´erez-D´ıaz et al.^17^ introduced a versatile approach for identifying and mitigating both low-rate and high-rate DDoS attack traffic within SDN networks, exhibiting behavior akin to regular traffic. The methodology relies on Intrusion Detection and Prevention Systems (IDS and IPS). Utilizing six machine learning models for DDoS classification within the IDS, a notable limitation emerges: the method incorporates a fixed threshold, rendering it ineffective in detecting low-rate DDoS attack traffic targeting multiple victims.

In contrast, our model offers several advantages for DDoS attack detection in SDN networks. Firstly, it provides flexibility in detection by dynamically adjusting clusters based on entropy values, enabling the identification of varying attack intensities and patterns, including low-rate attacks targeting multiple victims. Secondly, it facilitates the identification of anomalies in network traffic patterns by clustering traffic based on entropy values, allowing for the detection of deviations from normal behavior indicative of potential DDoS attacks. Additionally, k-means clustering is scalable and computationally efficient, making it well-suited for real-time DDoS detection in large-scale SDN networks. Finally, the model's adaptability to evolving threats is enhanced using entropy-based features and clustering, enabling it to adjust to changing attack strategies and network conditions over time.

### Machine learning strategies for DDoS detection and mitigation in SDN

Machine learning is a powerful technique that can be used for DDoS attack detection and mitigation. It can analyze large volumes of network traffic data and learn to identify patterns and anomalies that are associated with DDoS attacks^18^.

Li et al.^19^ introduced a DDoS detection model leveraging a support vector machine (SVM) algorithm, utilizing packet-in messages to acquire traffic data and extracting essential features such as the source IP address. While the model demonstrates high efficiency, its reliance on a small number of features may not adequately capture all attack behaviors. This limitation becomes apparent in scenarios where attackers embed malicious code within payload packets, leading to reduced accuracy in detecting application-level attacks. As a result, while the model efficiently processes traffic data, its effectiveness in detecting sophisticated DDoS attacks targeting application layers may be compromised.

Ye et al.^20^ developed an SVM classifier-based DDoS detection strategy. The suggested detection methodology involved gathering flow status information, extracting features, and categorizing the collected feature values. Additionally, the authors created a feature extraction module to extract DDoS attack-related features for the classifier to be trained, and they used flood-based attack traffic to show how well the suggested approach worked. In comparison to previously suggested methodologies, the strategy was proven to offer a high detection accuracy and a decreased false alarm rate. However, it should be emphasized that all the previously mentioned techniques suffered from the issue of unidentified real detection effects, in addition to the previously mentioned need for manually extracting a sizable number of features for training.

Cui et al.^21^ introduced a new approach for detecting and defending against DDoS attacks, grounded in cognitive-inspired computing and focusing on entropy analysis. Their method utilized switch flow table data to evaluate the entropy of data packet flows, particularly focusing on source and destination IP addresses. Through training a support vector machine (SVM), the system could generate specific DDoS attack detection modes, thereby enhancing its capability to identify and respond to different attack patterns. This cognitive-inspired computing strategy not only utilized advanced machine learning techniques but also emphasized the significance of entropy analysis in DDoS detection and mitigation. By incorporating entropy considerations into packet flow analysis, their technique provided a promising avenue for enhancing the effectiveness and adaptability of DDoS detection and defense mechanisms within network environments.

In contrast, the proposed model presents a more automated, scalable, and adaptable approach to DDoS attack detection, potentially overcoming limitations associated with SVM classifier-based strategies.

Hannache et al.^22^ developed a cutting-edge Neural Network-based Traffic Flow Classifier (TFC-NN) aimed at real-time detection of DDoS attacks. Their model was trained and implemented using a dataset comprising both regular and malicious traffic, and it was deployed within a real SDN architecture. Impressively, the TFC-NN achieved a remarkable global accuracy rate of 96.13%, showcasing its effectiveness in accurately discerning between normal and malicious network activity.

In another study, Cui et al.^23^ utilized clustering technologies such as K-means to identify malicious traffic within network streams. They further evaluated the efficacy of their approach by assessing communication latency, detection accuracy, and defense effectiveness. By leveraging packet-in message registers to filter out malicious traffic, they demonstrated the practical applicability of their scheme in real-world network environments.

Furthermore, Gu et al.^24^ introduced a sophisticated hybrid feature selection-based, semi-supervised K-means detection technique. This approach not only addressed the challenges posed by outliers and local optimality but also incorporated an enhanced density-based initial cluster center selection procedure. By integrating feature selection strategies and semi-supervised learning techniques, they aimed to enhance the robustness and accuracy of DDoS attack detection within SDN networks, highlighting the importance of advanced methodologies in mitigating cyber threats effectively.

### Deep learning for DDoS detection and mitigation in SDN

Deep learning (DL) has shown significant promise in the field of DDoS attack detection and mitigation. DL techniques, particularly neural networks (NNs), can effectively analyze large and complex datasets, learning intricate patterns and adapting to evolving attack strategies^25^.

In comparison to traditional machine learning (ML) techniques, deep neural network (DNN)-based solutions offer superior accuracy in identifying network traffic anomalies^26^. By leveraging sophisticated architectures and advanced learning algorithms, DNNs can effectively capture complex patterns and relationships within data. However, these solutions encounter challenges when dealing with large input dimensions and data volumes due to issues such as dimensionality and gradient diffusion. The gradient descent algorithm, commonly used in training DNNs, may struggle to converge efficiently in high-dimensional spaces, leading to longer training times and increased computational complexity. Furthermore, the heightened processing time and resource usage associated with analyzing network traffic data can impede the efficiency of DNN-based detection systems, particularly in real-time scenarios where rapid response is essential. Despite these challenges, the superior accuracy offered by DNNs underscores their potential to enhance network security and anomaly detection capabilities in various contexts.

Using the CICIDS 2017 dataset, Makuvaza et al.^27^ created a DNN-based technique for real-time DDoS threat detection in SDN. Using less time and resources, the algorithm detects DDoS threats with a 97.59% accuracy rate. Additionally, the authors suggested a two-level security detection system based on Snort, DNN, and SVM techniques. The DNN approach was tested and trained using the KDD Cup 1999 dataset, and results showed that it was more accurate than SVM.

For detecting slow DDoS attacks in the SDN environment, a deep learning framework has been created by Nugraha et al.^28^. The model gathers and analyses traffic flow statistics using the SDN controller’s REST API. Convolutional neural network-long short-term memory (CNN-LSTM) is the basis of the model. The model performs better than other deep learning models like the Multilayer Perceptron and 1-Class Support Vector Machine, according to experimental findings. The technique, though, is time-consuming and expensive for real-time detection.

Doriguzzi-Corin et al.^29^ introduced LUCID, a novel DDoS detection system designed to address computational constraints by using a one-dimensional convolutional neural network (CNN). Unlike traditional deep learning approaches, LUCID prioritizes efficiency without sacrificing accuracy. Experimental evaluations conducted on diverse datasets, including ISCX2021, CIC-IDS2017, and CSE-CIC2018, showcased remarkable results. LUCID exhibited a significant 40 × reduction in processing time compared to alternative deep learning techniques, making it highly suitable for deployment in resource-constrained environments. This efficiency enhancement underscores the system's potential to revolutionize DDoS detection by providing rapid and reliable protection against malicious attacks while minimizing computational overhead.

Liang et al.^30^ employed long short-term memory (LSTM) within their DDoS detection framework, leveraging the capabilities of recurrent neural networks (RNNs) to capture intricate temporal dependencies inherent in network traffic data. LSTM networks, equipped with three gating units, excel in modeling sequential data by selectively retaining and updating information over time. This feature enables LSTM to effectively capture the implicit sequence representations present in input vectors, making it well-suited for analyzing time-series data such as network traffic patterns. Unlike conventional methods that rely on labor-intensive and error-prone feature engineering, LSTM-based approaches facilitate automated learning of flow-level patterns directly from raw data. This automation not only enhances detection accuracy but also streamlines the development process by eliminating the need for manual feature extraction, thereby paving the way for more efficient and reliable DDoS detection systems.

Cil et al.^31^ introduced a traffic classification model utilizing deep neural networks (DNNs) to enhance accuracy in network traffic analysis. The model features a streamlined architecture with feature extraction stages, requiring only three fully connected layers for training completion. Experimental evaluation on the CIC-DDoS2019 dataset demonstrated impressive accuracy, reaching 95%. This highlights the effectiveness of DNN-based approaches in accurately classifying network traffic patterns, promising improved network security and anomaly detection capabilities. Moreover, the model's simplicity and high accuracy suggest its potential for practical deployment in real-world network environments, offering a valuable tool for enhancing network monitoring and defense systems.

A fully connected (FC) architecture was used in an explainable DL framework for the Industrial Internet of Things (IIoT) developed by Khan et al.^32^. An autoencoder-based detection framework that used convolutional and recurrent networks was proposed to identify cyber threats in IIoT networks. To enhance the learning of data features, the framework used a two-step sliding window (SW) method to extract temporal and spatial characteristics for attack event categorization and explanation. When compared to modern techniques, the empirical results showed how well the framework extracted contextual elements of dangerous patterns and how robust it was in identifying malicious events. The model performed better than state-of-the-art techniques, incorporated explanation mechanisms for model decisions, and presented an inventive approach for addressing cyber threats in IIoT networks. Additionally, Khan et al.^33^ introduced a deep-autoencoder-based intrusion detection system (IDS) for IIoT networks. The model, based on an LSTM auto-encoder design, accurately identified invasive events in real time. The model outperformed existing methods, achieving accuracy rates of 97.95% and 97.62% on benchmark datasets like the gas pipeline and UNSWNB-15.

Khan et al.^34^ also proposed a privacy-conserving intrusion detection framework named PC-IDS, tailored for securing Contemporary Smart Power Systems (SPNs) against cyber-attacks. The model used a hybrid machine learning approach, transforming raw data into a privacy-preserving format, and identifying malicious events using a probabilistic neural network. The framework outperformed existing methods in terms of false positive rate, detection rate, and computational processing time, enhancing SPN security and privacy. The framework achieved detection rates of 96.03% and 95.91%, demonstrating its effectiveness in enhancing SPN security. Additionally, Khan et al.^35^ highlighted the importance of protecting industrial control systems (ICSs) against cyber-attacks due to their integration with IoT technologies. The paper proposed a novel intrusion detection system (IDS) model called federated-simple recurrent units (SRUs) for IoT-based ICSs. The model used simple recurrent units’ architecture to mitigate computational costs and address gradient vanishing issues. Experimental validation showed that the model accurately detected intrusions in real-time without compromising privacy or security.

In comparison, the proposed model offers scalability, efficiency, and real-time detection. It can also adapt to changing network conditions and quickly identify anomalies, reducing processing time and resource usage. The entropy-based model with k-means clustering balances accuracy and efficiency, allowing for timely detection without significant processing delays.

### Blockchain for DDoS detection and mitigation in SDN

Blockchain technology has been proposed as a potential solution for DDoS detection and mitigation^36^.

Bose et al.^37^ devised a novel strategy to block DDoS attacks at the switch stage by incorporating blockchain-based encryption into the interaction channels between data and control planes. This innovative approach aims to enhance the security and resilience of SDN switches against malicious activities. Through simulation, the study illustrated the efficacy and reliability of employing blockchain technology as a defense mechanism, showcasing its potential to fortify SDN infrastructure against attackers. However, despite its promising benefits, the system exhibited certain limitations, notably unauthorized access, and data leakage, which emerged as significant drawbacks. Addressing these concerns is crucial for ensuring the comprehensive security and integrity of the SDN environment, underscoring the need for further refinement and optimization of blockchain-integrated defense mechanisms.

A confidence evaluation system with a focus on SDN and blockchain was introduced by Mathieu et al.^38^. The solution demonstrates how the SDN reduces surface attacks on the household network. The end user struggles with judgment and lacks the requisite abilities. The SDN architecture requires an appropriate access control mechanism for multiple organizations, including SDN controllers and switches. To address this issue, Durbadal et al.^39^ present blockchain-based access control. The outcome demonstrates that the Blockchain-based Access Control SDN (BACC-SDN) safeguards against possible threats, which is essential for an SDN network’s security. Addressing these limitations is crucial for effective integration into DDoS detection and mitigation strategies.

In comparison to existing studies in the field, the proposed model, which integrates system entropy with machine learning clustering techniques for DDoS detection and mitigation in SDN networks, offers several significant differentiators.

- Integration of System Entropy: While many articles only discuss statistical or ML techniques for DDoS detection, the suggested model incorporates system entropy as a fundamental element. A measure of the network's disorder, system entropy sheds light on how network traffic typically behaves. The model improves its capacity to recognize anomalies indicative of DDoS attacks by adding entropy analysis to the detection procedure, resulting in more precise and rapid detection.
- Real-Time Detection and Mitigation: Enabling real-time DDoS attack detection and mitigation is one of the main objectives of the proposed model. The model can detect anomalies and start mitigation measures promptly by continuously monitoring system entropy and applying ML clustering algorithms. This real-time responsiveness is essential for minimizing the impact of DDoS attacks on network availability and performance.
- Reduced False Positives: In DDoS detection, false positives are a common challenge that can result in needless alert fatigue and resource usage. By integrating machine learning clustering techniques with system entropy, the suggested model aims to address this issue. By analyzing several aspects of network traffic and detecting abnormal behavior clusters, the model can lower false positives and enhance DDoS detection precision.
- Experimental Validation: Experimental assessment of modern SDN environment-specific datasets, including CIC-IDS2017, CSE-CIC-2018, and CICIDS2019 strengthens the suggested model. These experiments show the model's practical application and performance by offering empirical evidence of its ability to identify and mitigate DDoS attacks in real-world scenarios.

## Methods

This section introduces the proposed scheme. It describes the overall design of the proposed comparative scheme, including the entropy-based, machine learning algorithms employed, and the system’s main parameters.

**Figure 2:**
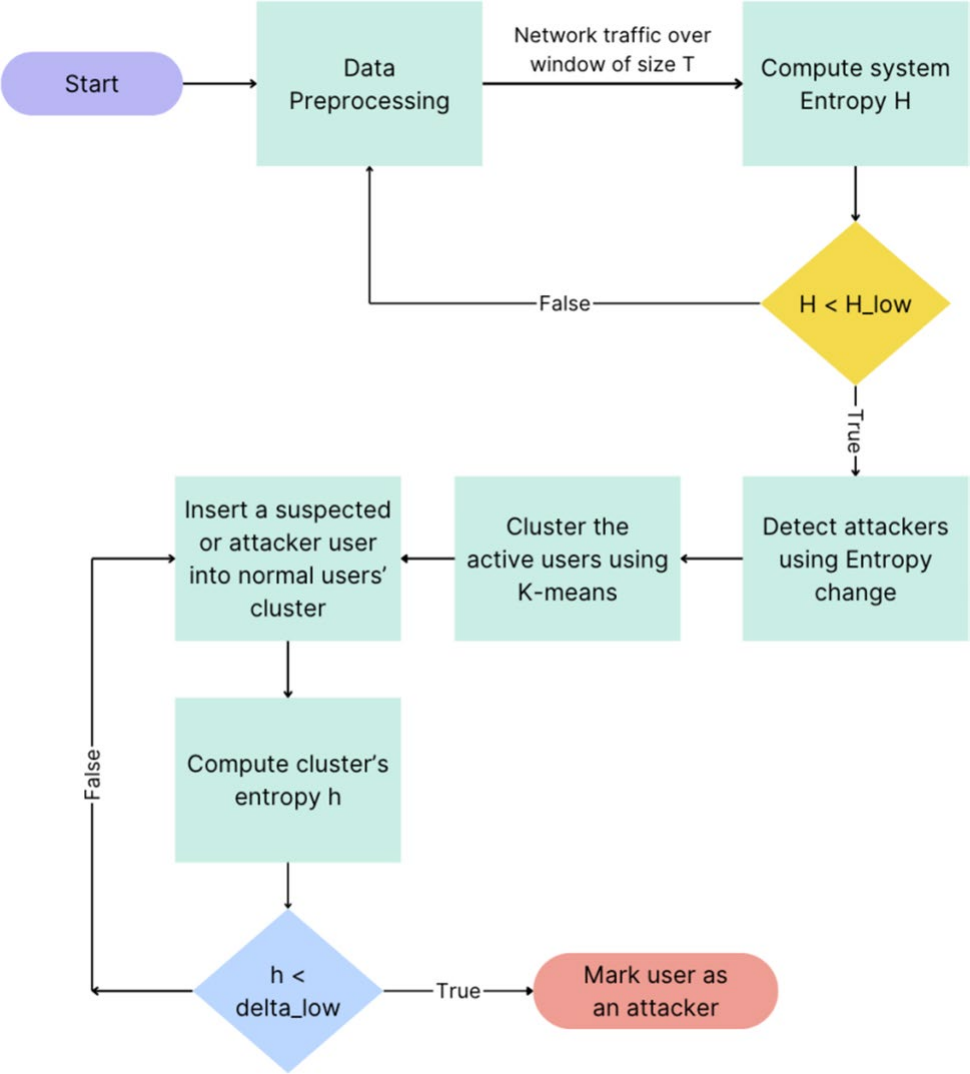
Model architecture.

The proposed scheme consists of entropy-based and machine-learning clustering modules, strategically integrated to enhance the effectiveness of DDoS attack detection and mitigation. Figure 2 illustrates the comprehensive architecture of this model. The primary step involves data preprocessing which is performed through the following sequence:

1. Each dataset has different features representing the requests, mostly:
  $$ ip_{{src}} ,\;port_{{src}} ,\;port_{{dest}} ,\;protocol,\;time,\;label,\;pktSiz{\text{e}} $$
  where ${ip}_{src}$, *ip* are ${ip}_{dest}$, addresses of both source and destination and *pktSize* is the packet size.
2. Extract important features from the dataset based on flow-based feature extraction technique. This technique aims to analyze how features are distributed across traffic packets^40^, like
  $$ ip_{{src}} ,\;port_{{src}} ,\;port_{{dest}} ,\;protocol,\;time,\;label $$
  These network flow attributes enable packets, whether normal or anomalous, to be specified.
  The entropy values are then compared to a preset threshold to locate the presence of anomalies in these features.
3. Clean data by removing any missing information.
4. Grouping:
  Choose interval length T.
  Choose *uid* to be an identifier of each user, with representation.
    $${ip}_{src}$$−$${ip}_{dest}$$ for example,
  Group requests in the same interval from the same *uid* as follows: *interval*#, *uid*, *count*, *label*.
    Where *count* is the number of requests of the user defined by *uid* within the interval.

Subsequently, the request flows are divided into multiple time intervals of equal duration, denoted as T. After each time interval, the entropy detection module comes into play, assessing whether an attack occurred in the last interval. In the case of a detected attack, the machine learning technique starts the clustering of active users within that interval into three distinct clusters: normal users, suspicious users, and attackers. This sequential process emphasizes the model's dynamic responsiveness to potential attacks, integrating both entropy-based detection and machine learning clustering for a comprehensive defense mechanism.

### Entropy detection module

After each time interval, the frequencies, representing the total attempt requests of active users within that interval, go through a normalization process. This normalization, shown by the following equation, ensures that the frequencies are scaled appropriately. In this equation, ${\widehat{\text{x}}}_{\text{i}}$ is the normalized frequency of user i, ${\text{x}}_{\text{i}}$ represents the frequency of user i within set X, ${\text{x}}_{\text{min}}$ is the minimum frequency in set X, and ${\text{x}}_{\text{max}}$ denotes the maximum frequency in set X. This normalization step is instrumental in maintaining consistency and relevance in the dataset, facilitating a robust analysis of user behavior, and aiding subsequent detection processes.

$${\widehat{x}}_{i}= \frac{{x}_{i}- {x}_{min} }{{x}_{max}- {x}_{min}}$$

Entropy is a scientific concept, as well as a measurable physical property, associated with disorder, randomness, or uncertainty. A fundamental assumption is introduced, that the system's randomness decreases during a DDoS attack. After each time interval, the system's overall entropy is calculated using the following equation. In this equation, entropy reaches its maximum when all elements have the same frequencies, and subsequently decreases as the randomness of requests decreases. Here, X represents the set of active users in the interval, and ${\text{x}}_{\text{i}}$ ${\text{x}}_{\text{i}}$ is the frequency of user $\text{i}$ requests in the interval. This entropy calculation serves as an essential metric for calculating the level of randomness and potential irregularities in the system, thereby helping in the identification of DoS attacks.

$$H\left(X\right)\leftarrow -{\sum }_{{x}_{i}\in X}^{\infty }p(xi) \times log(p({x}_{i}))$$

**Figure 3:**
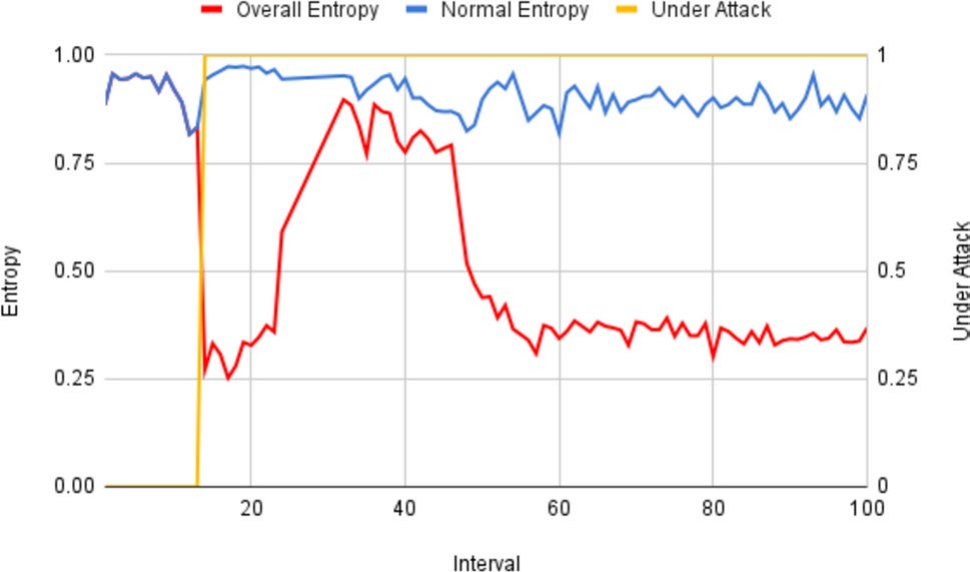
Entropy for the system during DDoS and entropy for the normal users in the same intervals on the CIC-IDS2017 dataset.

The module sets a lower threshold ${\upbeta }_{\text{lower}}$ for the system’s overall entropy H(X), which indicates that the system is under attack. Figure 3 shows the system entropy during the DDoS attack and the entropy of the normal users only in the same intervals on a part of the CIC-IDS2017 dataset with an interval size of 120 s. The figure shows a noticeable decrease in entropy after the DDoS attack started, yet sometimes both entropy values were near each other, possibly due to having slow attack rates. This interaction between the system and normal user entropy provides valuable insights into the changes in the DDoS attack, particularly during intervals marked by subtle variations in attack intensity.

The first procedure, the attack detection has O(n) time complexity and O(n) space complexity, where n is the number of users in the interval. The detection procedure consists of a clustering procedure and an attacker detection procedure. The clustering has a time complexity of O ($K \times n \times I$) where k is the number of clusters (3 in our case) and I the number of iterations which is fixed in our case, so overall the clustering has O(n) time complexity and O(n) space complexity. The attacker detection procedure has O(n) time complexity (using Algorithm 3.2). Therefore, the overall time complexity for the system is O(n) and O(n) space complexity, where n is the number of active users in the interval.

Algorithm 3.1 encapsulates the comprehensive detection procedure, offering a brief overview of the steps involved in identifying potential attackers within the system.

**Figure:**
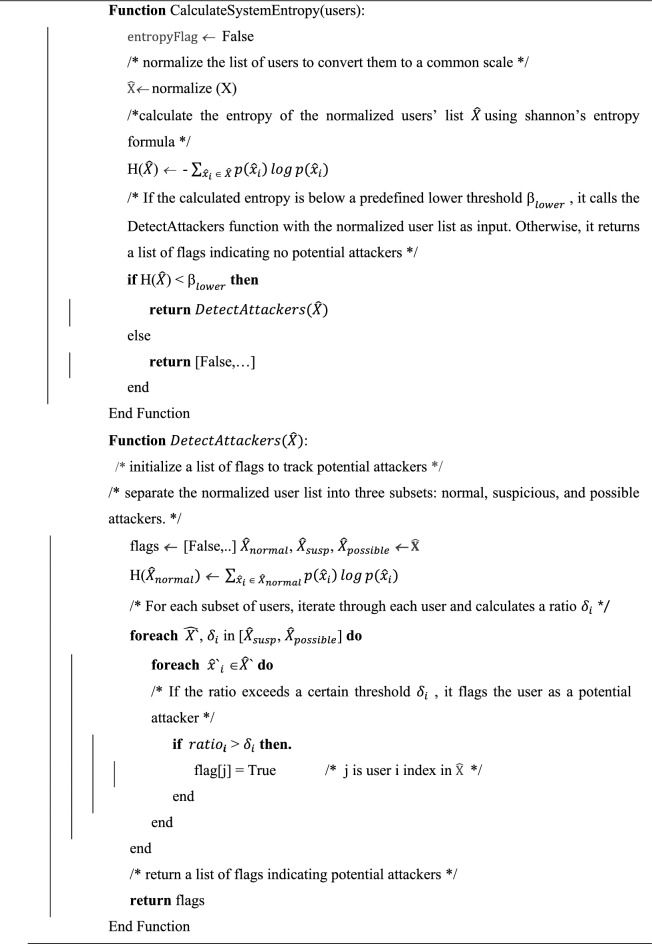
Algorithm 3.1: Detection Procedure

Algorithm 3.2 provides a detailed illustration of the applied optimization method, specifically designed to compute.

H (X ∪ ${u}_{j}$) This optimization serves the purpose of significantly reducing computational complexity, transitioning from O (${n}^{2}$) to a more efficient O ($n$). This strategic enhancement contributes to the algorithm's streamlined efficiency, ensuring a more scalable and expedited process for calculating entropy.

**Figure:**
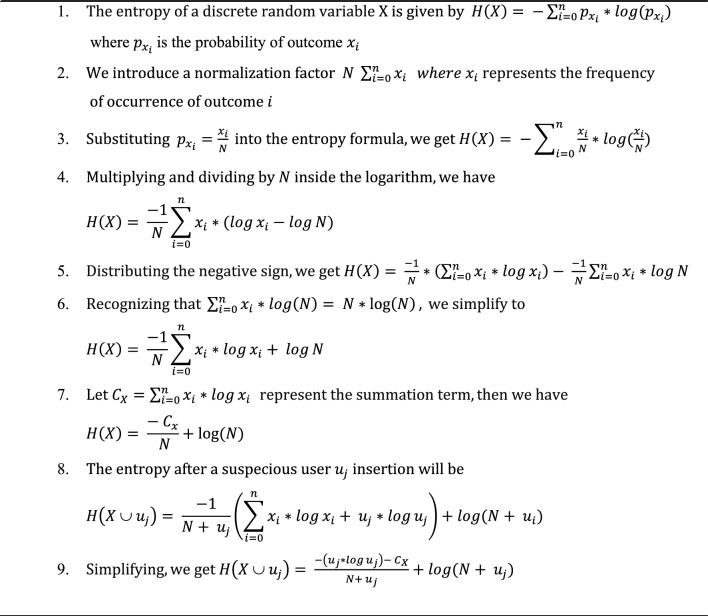
Algorithm 3.2: Entropy Calculation Improvement

### Machine learning clustering module

**Figure 4:**
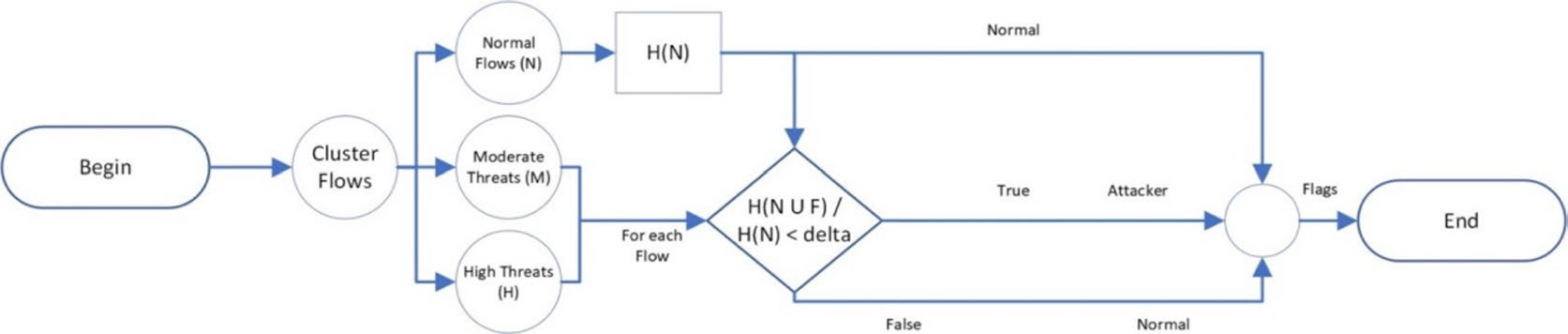
Machine learning clustering module.

Following the detection of an attack by the entropy module within the system, the subsequent step involves the application of the K-means algorithm to cluster active users from the previous interval. These users are clustered into three distinct groups: normal users, suspicious users, and potential attackers, as shown in Fig. 4. The utilization of the K-means clustering method is attributed to its versatility and efficiency in handling substantial volumes of data promptly. The algorithm's effectiveness lies in its ability to efficiently group network traffic data points into clusters, making it suitable for the rapid and real-time identification of user behavior patterns. However, a key challenge in employing K-means is the selection of an appropriate value for "k", representing the number of clusters. Selecting an unsuitable "k" value can lead to suboptimal clustering outcomes.

To address this challenge, we have opted for a value of "k" equal to 3, aligning with the need to classify users into three primary clusters. This decision ensures that the K-means algorithm effectively captures the nuances of user behavior and facilitates the differentiation of normal users, suspicious users, and potential attackers within the system.

After clustering the users into the three clusters, the evaluation process proceeds to test each user (${\text{u}}_{\text{j}})$ within the suspicious and potential attackers' clusters against the normal users' cluster. This examination aims to assess the impact of each user on the entropy of the normal users' cluster, as expressed in the following equation. Within the examined clusters, distinct thresholds are established for the entropy ratio change, denoted as Suspicious Cluster Entropy Change Delta (${\updelta }_{\text{susp}}$) and Attacker Cluster Entropy Change Delta (${\updelta }_{\text{attack}}$). The evaluation criterion involves comparing the ${\text{ratio}}_{\text{i}}$ (h) with the corresponding ${\updelta }_{\text{i}}$ for each cluster. If the ratio change is less than the predetermined threshold ${\updelta }_{\text{i}}$ (${\text{ratio}}_{\text{i}}$ (h) < corresponding, ${\updelta }_{\text{i}}$), the user is identified as a potential attacker. This demanding assessment process ensures a robust and accurate identification of suspicious and potential attacker users within the system.

$${\text{ratio}}_{\text{i}}(\text{h})= \frac{\text{H}(\text{X }\cup {\text{ u}}_{\text{i}})}{\text{H}(\text{X})}$$

### Training parameters

The system includes a variety of parameters (data attributes) that manage its interaction with incoming request flows. These parameters collaboratively influence the system's performance, serving a crucial role in guaranteeing optimal functionality and responsiveness. A summary of these parameters is described in Table 1:

**Table 1:** System parameters.

Parameter	Description
Interval Size	The duration after which the system considers one interval done
Entropy lower bound threshold (${\beta }_{lower}$)	The system entropy below which entropy considers the system to be under attack
Suspicious cluster entropy change delta (${\delta }_{susp}$)	The entropy ratio below which the system considers the suspected user as an attacker
Attacker cluster entropy change delta (${\delta }_{attack}$)	The entropy ratio below which the system considers the possible attacker as an attacker when it is tested against the normal users’ k-means clustering group

In this study, we focused on four main parameters, namely interval size, ${\upbeta }_{\text{lower}},$ ${\updelta }_{\text{susp}}$, and ${\updelta }_{\text{attack}}$. From self-assessment on CIC-IDS2017^41^, the four parameters presented similar behavior concerning system accuracy. Initially, accuracy starts increasing with the increase of the respected parameter until it reaches its peak and then starts decreasing with the continuousincrease in the parameter value. This pattern mirrors the characteristic curve observed in normal distribution patterns.

## Evaluation scheme

This section provides a comprehensive overview of the evaluation environment employed in this study. It includes an extensive exploration of the datasets utilized. To ensure a comprehensive and open discussion about the results, it also specifies the exact evaluation criteria that will be used to evaluate the effectiveness of the suggested approaches.

### Datasets description

*CIC-IDS2017* dataset has been curated by the Canadian Institute for Cybersecurity (UNB) to accurately describe network activities and simulate attack scenarios based on real security reports. The dataset offers a comprehensive range of network behaviors for analysis. It includes various protocols like Hypertext Transfer Protocol (HTTP), Secure Shell Protocol (SSH), File Transfer Protocol (FTP), Hop-by-Hop IPv6 (HOPOPT), Transmission Control Protocol (TCP), and User Datagram Protocol (UDP). The refined data preserves key characteristics like Source IP, Destination IP, Source Port, Protocol, Timestamp, and Label and simplifies it for efficient model evaluation^41^.

*CSECIC2018* dataset is a crucial tool for cybersecurity researchers, providing network traffic data for intrusion and DDoS detection models and algorithms. Like CIC-IDS2017, this dataset covers various protocols, offering a comprehensive view of network behaviors, including HTTP DDoS attacks^42^.

*CICDDoS2019* analyzes DDoS attacks, including both benign and common ones. The dataset enables the analysis of network traffic and the extraction of over 80 traffic features. It features labeled flows categorized by timestamp, source and destination IPs, source and destination ports, protocols, and attack types^43^.

Table 2 provides a summary of the three datasets. The model assessment was carried out on CIC-IDS2017^41^ dataset.

**Table 2:** Summary of datasets.

Dataset	Size	Duration	Format	Publicly available	Contains attack traffic	Attack types
CICIDS2017^41^	50 GB	5 days	Packet	yes	yes	DoS, DDoS
CSECIC2018^42^	500 GB	2 days	Packet	yes	yes	Brute force, DoS, Botnet, DDoS attacks
CICDDOS2019^43^	150 GB	2 days	Packet	yes	yes	Modern reflective DDoS attacks such as DNS, NetBIOS, LDAP, MSSQL, UDP, UDP-Lag, SYN, NTP, DNS, SNMP, and WebDDoS

### Evaluation metrics

To conduct a comprehensive performance evaluation, it is essential to consider a variety of metrics. The confusion matrix is used to evaluate the performance of the model. The performance assessment is done through four main measures as follows: true positive (TP), true negative (TN), false positive (FP), and false negative (FN). Where the positive indicates being an attacker. The system accuracy is calculated as the number of correct labeling as shown in the subsequent equation.

$$Accuracy= \frac{TP + TN}{TP + TN + FP+ FN}$$

In our model, accuracy (acc) and the other confusion metrics are calculated for the mitigation effect, in other words, if the entropy detected that the user is an attacker, the TP value will be affected in the next interval since all the requests in the current interval were handled (i.e., FN).

In addition to the accuracy, false positive rate (FPR), Precision, Recall, and F1-Score are calculated for the technique as the following. Recall (sensitivity) measures the percentage of attackers that are correctly detected. Additionally, F1-score symmetrically represents both recall and precision.

$$FPR= \frac{FP}{FP+TN}$$

$$Precision \left(prec\right)= \frac{TP}{TP+FP}$$

$$Recall (recall)= \frac{TP}{TP+FN}$$

$$F1Score=\frac{2 \times prec \times recall}{prec + recall}$$

One of the main challenges when utilizing datasets for training and testing machine learning models, including those considered for evaluating the proposed approach, is data imbalance. Data imbalance can cause machine learning algorithms to prioritize accuracy for the majority class while neglecting the minority class, resulting in biased models. To make sure that the model can effectively identify patterns from all classes, dataset imbalance must be addressed. Dataset dimension reduction can help address data imbalance by mitigating the impact of the majority class dominating the feature space^44^. Performance measures such as Matthews Correlation Coefficient (MCC), and Geometric Mean (G-means) are frequently used to evaluate how well machine learning models perform, especially when dealing with imbalanced datasets. MCC considers true and false positives and negatives and ranges from − 1 to 1. A coefficient of 1 represents a perfect prediction, 0 represents a random prediction, and − 1 indicates total disagreement between prediction and observation.

On the other hand, G-means evaluates the classifier's performance by considering both sensitivity and specificity. Sensitivity measures the classifier's accuracy in detecting stuck-up events, while specificity evaluates its ability to identify non-stuck-up events, providing a balanced assessment of classification accuracy^45^. These metrics provide a more comprehensive assessment of the model’s performance, enabling more accurate decision-making in real-world applications.

$$MCC= \frac{TP\times TN-FP\times FN}{\sqrt{\left(TP+FP\right)(TP+FN)(TN+FP)(TN+FN)}}$$

$$G-means= \sqrt{ sensitivity\times specificity }=\sqrt{ \frac{TP}{TP+FN}\times \frac{TN}{TN+FP}}$$

## Results

This study conducts a series of experiments, including both self-assessment and comparative analyses. In the beginning, a single-protocol dataset generator was implemented, to be as an initial phase to test the sensitivity of the system parameters. After that, the system parameters go through comprehensive testing against the CIC-IDS2017^41^ dataset. Finally, the system's performance was thoroughly assessed against multiple datasets, including CIC-IDS2017^41^, CSECIC2018^42^, and CIC-DDoS2019^43^. The system parameters’ sensitivity was used to plan the training approach used later. The rest of this section illustrates the results of both the self-assessment and comparative analyses, detailing the various phases of our methodology and the outcomes observed at each step.

### Model assessment

The proposed model contains multiple parameters that control its results, as shown in "Methods" section. In this work, we focused on the following parameters.

- Interval Size
- Entropy Lower Threshold (${\upbeta }_{\text{lower}})$
- Possible Attackers entropy Delta (${\updelta }_{\text{attack}})$
- Suspicious Users entropy delta (${\updelta }_{\text{susp}})$

In the beginning, the model is trained at a selected interval size (mostly 120-s intervals). After that, each parameter has a value range depending on its type, for the entropy lower threshold, entropy deltas [0, 1].

The experiments reveal distinct phases in the behavior of the key parameters ${\upbeta }_{\text{lower}}$, ${\updelta }_{\text{attack}}$, and ${\updelta }_{\text{susp}}$, each affecting the system's accuracy and false positive rate (FPR) differently. For instance, varying ${\upbeta }_{\text{lower}}$ shows three distinct phases, with the system transitioning from low accuracy to high accuracy as it is adapted to different entropy ranges. Similarly, ${\updelta }_{\text{attack}}$ shows three phases, with its impact on accuracy being less significant compared to ${\upbeta }_{\text{lower}}$, but still contributing to overall system performance.

#### CIC-IDS2017 self-assessment

**Figure 5:**
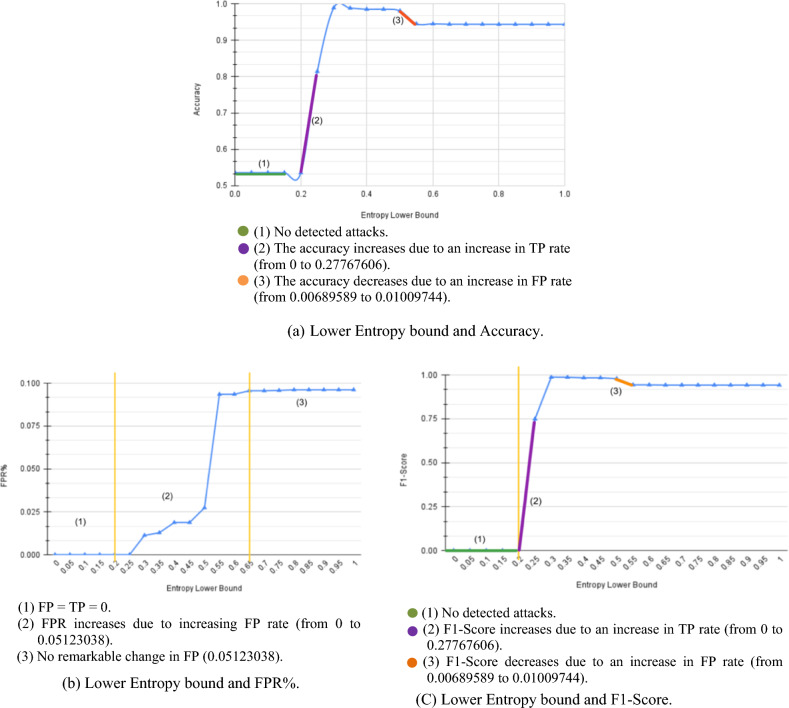
CIC-IDS2017 ${\beta }_{lower}$ results.

A detailed self-assessment of our model is conducted using CIC-IDS2017^41^ dataset. Experiments measure the impact of each attribute ${\upbeta }_{\text{lower}}$, ${\updelta }_{\text{attack}}$, and ${\updelta }_{\text{susp}}$ on metrics such as accuracy, FPR, and F1-Score. Figure 5a shows the results of changing the value of ${\upbeta }_{\text{lower}}$ where the accuracy is the lowest with FPR = 0 since the entropy detection alarm is never fired and thus no additional checks are carried out. Then, the accuracy starts to increase significantly after ${\upbeta }_{\text{lower}}$ = 0.2, and this can be justified by Fig. 3 where the entropy of all attacking intervals H(U) > 0.25, so ${\upbeta }_{\text{lower}}\hspace{0.17em}$< 0.25 won’t cause any checks. After ${\upbeta }_{\text{lower}}\hspace{0.17em}$= 0.2, with the increase in ${\upbeta }_{\text{lower}}$ value, the system starts inspecting more intervals and detecting more attackers, which increases the TP rate and accuracy till it reaches a certain limit. With the continuous increase of ${\upbeta }_{\text{lower}}$, the system starts considering normal intervals as under-attack intervals, and thus FP rate increases, and accuracy decreases due to considering high-rate normal users as attackers.

Regarding FPR, as shown in Fig. 5b, the curve can be divided into three phases: very low FPR with ${\upbeta }_{\text{lower}}\hspace{0.17em}$< 0.2 where FP = TP = 0, rapidly increased accuracy with rapid FP and FPR, and a remarkably increased FP affecting accuracy, resulting in higher FPR. Figure 5c shows that the F1-Score has observations as in accuracy.

Overall, the effect of ${\upbeta }_{\text{lower}}$ has three phases. The first phase, where ${\upbeta }_{\text{lower}}<H(U)$, H(U) is the minimum attacking interval’s entropy, is characterized by low FP and TP rates, thus a lower accuracy and F1-Score since the module doesn’t consider any interval suspicious. In the second phase, where ${\upbeta }_{\text{lower}}$ covers the range of suspicious intervals, causing an increase in accuracy due to the increase in TP. Finally, the third phase where ${\upbeta }_{\text{lower}}$ covers the normal intervals’ entropy ranges, where the accuracy slightly decreases due to considering high-rate users as attackers, i.e., FP increases.

**Figure 6:**
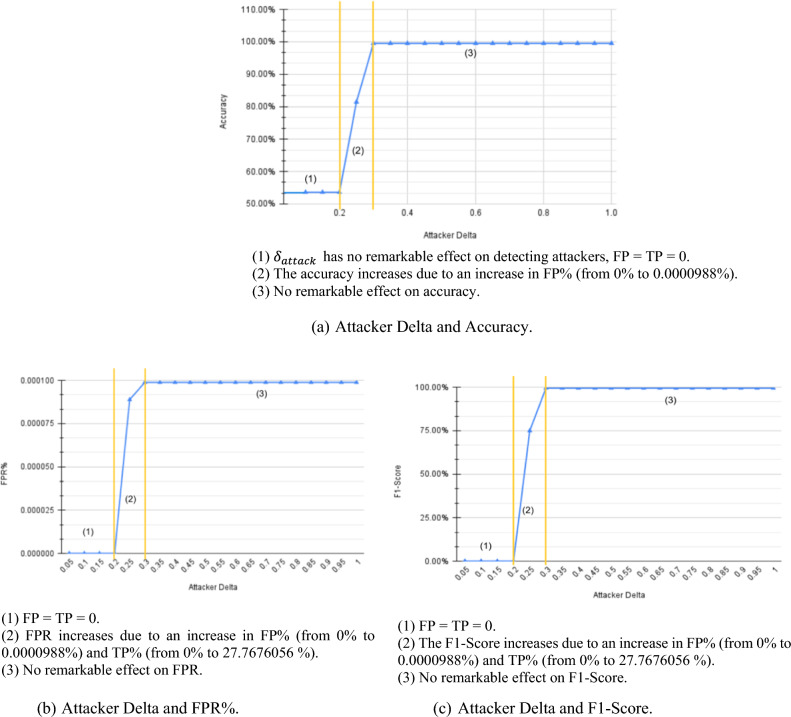
CIC-IDS2017 ${\delta }_{attack}$ results.

The study demonstrates a similar effect on the ${\updelta }_{\text{attack}}$ in three phases, as shown in Fig. 6. Figure 6a shows that at lower values, accuracy is very low since most of the attackers fall in the possible attackers’ cluster. As ${\updelta }_{\text{attack}}$ increase, more attackers are detected, increasing TP, FP, and accuracy. However, the impact of the ${\updelta }_{\text{attack}}$ on system accuracy is less remarkable compared to the effect of ${\upbeta }_{\text{lower}}$ value. In the third phase, increasing ${\updelta }_{\text{attack}}$ doesn't significantly impact accuracy due to the higher likelihood of the system being attacked.

The analysis shows nuanced relationships between the attributes (${\upbeta }_{\text{lower}}$, ${\updelta }_{\text{attack}}$, and ${\updelta }_{\text{susp}}$) and system performance. For example, lower values of ${\updelta }_{\text{attack}}$ lead to lower accuracy due to missed detections of attackers, while higher values increase false positives. These observed patterns help in understanding the behavior of the model under different conditions, guiding parameter tuning and training approaches for improved performance.

Overall, the effect of ${\delta }_{attack}$ can be divided into three phases. In the first phase, at low ${\delta }_{attack}$ values, the procedure will not detect attackers, i.e. very low TP, and thus very low accuracy and high FN. Followed by the second phase, where accuracy reaches a peak due to high TP and low FN rates. Finally, at high ${\delta }_{attack}$ rates, the FP starts increasing due to considering high-rate normal users as attackers, causing a slight decrease in accuracy and F1-score.

**Figure 7:**
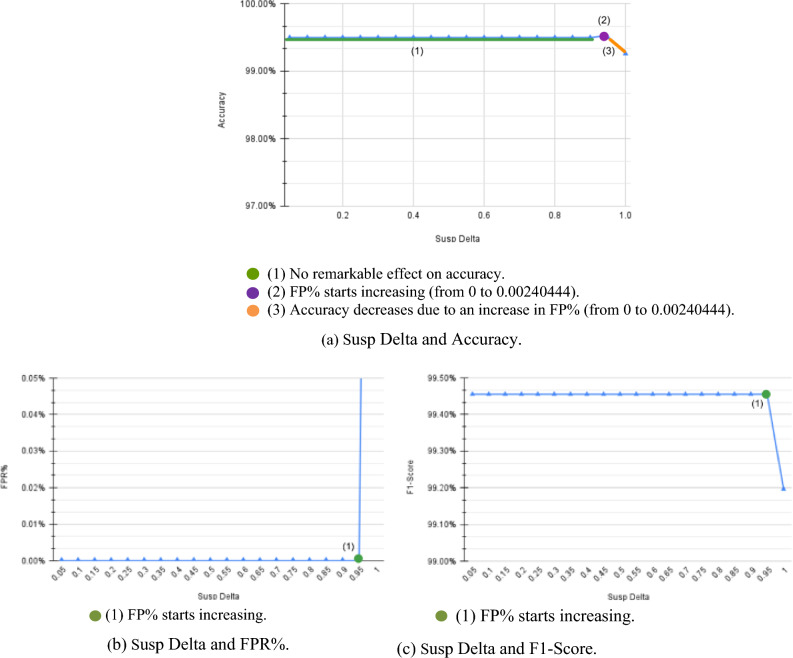
CIC-IDS2017 ${\delta }_{susp}$ results.

Finally, Fig. 7 shows the effect of ${\updelta }_{\text{susp}}$. There is no remarkable effect on the system. It seems that no true attackers fall in the suspicious cluster, so the system has lower accuracy with lower ${\updelta }_{\text{susp}}$ as shown in Fig. 7a. Only at higher values of ${\updelta }_{\text{susp}}$, the system starts to falsely mark normal users as attackers, increasing FP and FPR and decreasing the system’s accuracy and F1-Score.

The ${\delta }_{susp}$ was expected to have a similar pattern as the ${\delta }_{attacker}$, yet it showed no significant effect on the system, except at very high values, where it decreased accuracy due to an increase in FP. With further investigations, we found no attackers falling into this cluster in our datasets. Yet, it’s safer to consider checking the suspicious cluster to detect cases where there exists a very high-rate attacker that will change the cluster of lower-rate attackers resulting in some attackers falling into the suspicious cluster.

#### Benchmarking

The proposed system is evaluated using three distinct datasets: CICIDS2017^41^, CSECIC2018^42^, and CICDDOS2019^43^. Performance metrics such as accuracy, FPR, TPR, F1-Score, G-means, and MCC are used for comparison. The results of the evaluation are presented in Table 3. For each dataset, the system performance is compared to multiple techniques. The results will be discussed in the remainder of this subsection.

**Table 3:** Results on datasets with 90% training size.

Dataset	Accuracy	FPR%	TPR% (Recall)	F1-score	G-means	MCC
CICIDS2017	0.9994	0.0000	0.9993	0.9997	0.9997	0.7298
CSECIC2018	0.9992	0.0045	0.9992	0.9996	0.9973	0.7043
CICDDOS2019	0.9997	0.0023	0.9990	0.9992	0.9984	0.8127

In the context of the CICIDS2017^41^ dataset, the performance of the system is compared against other notable techniques, including Lucid^29^, Multi-layer Perception (MLP), 1D-CNN^46^, LSTM^46^, and the combined approach of 1D-CNN + LSTM^46^. This comparative analysis aims to provide a thorough understanding of how the proposed system fares against established methods, shedding light on its strengths and effectiveness in the specific context of the CICIDS2017^41^ dataset. The ensuing discussion will clarify these comparative results as shown in Table 4.

**Table 4:** Results on CICIDS2017.

Dataset	Accuracy	FPR%	TPR% (Recall)	F1-score
Proposed system	0.9994	0.0000	0.9993	0.9997
Lucid^29^	0.9967	0.0059	0.9994	0.9966
MLP	0.8634	NA	0.8625	0.8735
1D-CNN^46^	0.9514	NA	0.9017	0.9399
LSTM^46^	0.9624	NA	0.9474	NA
1D-CNN + LSTM^46^	0.9716	NA	0.9910	0.9825

Roopak et al.^46^ proposed four different DL models for DDoS attack detection in Internet of Things (IoT) networks. The models are built with combinations of LSTM, CNN, and fully connected layers. The input layer of all the models consists of 82 units, one for each flow-level feature available in CICIDS2017^41^, while the output layer returns the probability of a given flow being part of a DDoS attack. The model intelligent detection convolutional neural network (ID-CNN) + LSTM^46^ produces good classification scores, while the others seem to suffer from high FN rates. The same observations are found on the CICIDS2017^41^ dataset, the increase in FN and decrease in FP leads to an increase in accuracy compared to Lucid^29^, and eventually, the other techniques used. Then, the CSECIC2018^42^ dataset results were compared to the Lucid^29^ technique as shown in Table 5.

**Table 5:** Results on CSECIC2018.

Dataset	Accuracy	FPR%	TPR% (Recall)	F1-Score
Proposed system	0.9992	0.0045	0.9992	0.9996
Lucid^29^	0.9987	0.0016	0.9989	0.9987

The CSECIC2018^42^ showed different behavior than the other two datasets, the FP increased, and the FN decreased, but overall, the system has a simple improvement in accuracy due to the Lucid^29^ technique. The dataset had a flow with a high rate compared to other flows which caused this increase in FP rate since this flow was falsely detected as an attack.

Finally, the CICDDOS2019^43^ dataset results were compared to Cil et al.^31^ and Alghazzawi et al.^47^ techniques as shown in Table 6. The proposed system shows higher accuracy rates compared to Cil et al.^31^ and Alghazzawi et al.^47^. The system showed higher FP rates compared to CICIDS2017^41^.

**Table 6:** Results on CICDDOS2019.

Dataset	Accuracy	FPR%	TPR% (Recall)	F1-score
Proposed system	0.9997	0.0023	0.9990	0.9992
Cil et al.^31^	0.9457	NA	0.9515	0.8721
Alghazzawi et al.^47^	0.9452	NA	0.9204	0.9344

The proposed model consistently outperforms existing techniques across all datasets, demonstrating superior accuracy and robustness in DDoS attack detection. Comparative analysis with techniques such as Lucid^29^, MLP, and 1D-CNN^46^ highlight the strengths of our model, particularly in mitigating false positives and achieving high accuracy rates.

This study contributes to the advancement of DDoS detection methods by providing a comprehensive analysis of key system parameters and their impact on performance. The research outcomes establish the foundation for future investigations that aim to enhance the efficiency and effectiveness of DDoS detection systems in real-world scenarios.

## Conclusion and future work

This study examines the process of detecting DDoS attacks in SDN environments, highlighting the effectiveness of a hybrid methodology in detecting and mitigating these attacks, emphasizing its practicality and relevance. The key findings and observations of this paper can be summarized as follows:

1. The hybrid methodology proposed in this study effectively integrates statistical and machine-learning clustering techniques, offering a robust solution for detecting and mitigating DDoS attacks in SDN environments.
2. Integration of entropy-based alerting with clustering analysis empowers the hybrid method to effectively address sudden and rapidly initiated DDoS attacks, enhancing the system’s resilience against such attacks.
3. Integration of an entropy-based alerting and detection mechanism enables real-time monitoring of incoming requests, enabling rapid detection and mitigation of potential attacks and enhancing system responsiveness. Once an attack is suspected, the active users are clustered based on their request rates and filtered based on their influence on the system's entropy to quickly identify potential attackers.
4. Utilization of the K-means clustering algorithm provides an efficient means of analyzing the influence of active users on the system’s entropy, contributing to improved defense mechanisms against DDoS attacks.

Future research directions focusing on enhancing detection accuracy, exploring alternative methodologies, and addressing system optimization challenges can further strengthen the resilience of SDN networks against DDoS attacks. The future scope and implications of this study could include:

- Exploring the incorporation of more advanced machine learning algorithms to enhance the accuracy of users’ classification based on their behavior (request types) within the system.
- Evaluation of alternative clustering methods beyond K-means could provide valuable insights into optimizing system performance and scalability, providing a more comprehensive perspective on clustering techniques suitable for DDoS attack detection in SDN environments.
- Implementation of trust-management mechanisms for mitigating DDoS attacks could provide additional layers of security to the system. This method allows users to accumulate a trust score during their interaction with the system, which can be used to verify their legitimacy. If any abusive behavior occurs, the trust score will decrease, and if it falls below a certain threshold, the user will be blocked from further interactions with the system. Applying this approach could enhance overall security measures.
- Optimization efforts should be directed towards mitigating the time and space complexity introduced by the entropy detection mechanism. Effective system performance depends on optimizing the entropy detection procedure, which guarantees low computational overhead and resource consumption for smooth functioning in real-world SDN

## Data Availability

The datasets used and analyzed during the current study are publicly available as the following: CICIDS2017 dataset is available at https://www.unb.ca/cic/datasets/ids-2017.html, CSECIC2018 dataset is available at https://www.unb.ca/cic/datasets/ids-2018.html, and CICDDOS2019 dataset is available at https://www.unb.ca/cic/datasets/ddos-2019.html.
